# Multiple Regression Analysis Reveals MicroRNA Regulatory Networks in *Oryza sativa* under Drought Stress

**DOI:** 10.1155/2018/9395261

**Published:** 2018-10-04

**Authors:** Jiajia Chen, Liangzhi Li

**Affiliations:** School of Chemistry, Biology and Materials Engineering, Suzhou University of Science and Technology, No. 1 Kerui Road, Suzhou 215011, China

## Abstract

Drought is a major abiotic stress that reduces rice development and yield. miRNAs (microRNAs) are known to mediate posttranscriptional regulation under drought stress. Although the importance of individual miRNAs has been established, the crosstalks between miRNAs and mRNAs remain unearthed. Here we performed microarray analysis of miRNAs and matched mRNA expression profiles of drought-treated rice cultivar Nipponbare. Drought-responsive miRNA-mRNA regulations were identified by a combination of a partial least square (PLS) regression approach and sequence-based target prediction. A drought-induced network with 13 miRNAs and 58 target mRNAs was constructed, and four miRNA coregulatory modules were revealed. Functional analysis suggested that drought-response miRNA targets are enriched in hormone signaling, lipid and carbohydrate metabolism, and antioxidant defense. 13 candidate miRNAs and target genes were validated by RT-qPCR, hierarchical clustering, and ROC analysis. Two target genes (DWARF-3 and P0651G05.2) of miRNA coregulatory modules were further verified by RLM-5′ RACE. Together, our integrative study of miRNA-mRNA interaction provided attractive candidates that will help elucidate the drought-response mechanisms in *Oryza sativa*.

## 1. Introduction

Rice (*Oryza sativa*) is one of the main staple crops that feed more than half of the global population [1]. Rice is acclimated to rain-fed or fully irrigated fields and is susceptible to drought stress [2]. The yield of rice is frequently challenged by dwindling water resources. There is a growing need to find novel drought-responsive molecules to improve the rice cultivars [3].

miRNAs (microRNAs) are short noncoding RNAs that mediate gene expression by mRNA degradation or translational repression [4]. Currently, 757 mature rice miRNA sequences have been identified in miRBase release 22 [5].

miRNAs have emerged as important players in a wide range of biological processes in plants [6]. They are involved in the resistance to environmental stresses by regulating the expression of stress-responsive genes [7–12]. Over the past decade, high-throughput technologies, e.g., microarray hybridization and RNA-seq, have accelerated the transcriptomic studies and shed light into the miRNA repertoires under drought stress conditions [13]. Drought-specific miRNAs have been investigated in different crops, including wheat [14–16], barley [17], maize [18], and Brachypodium [19], and dehydration-responsive miRNA expression patterns were detected. Several drought-related miRNA families have been identified in rice [20–24].

Drought-responsive miRNAs regulate their target mRNAs, most of which are functional proteins involved in drought stress [25]. Therefore, the function of a miRNA is often inferred via its target genes [26]. However, the identification of miRNA target genes represents a combinatorial challenge. Early miRNA target prediction methods rely on sequence complementarities, conservation, free energy, or target accessibility. However, these tools usually predict hundreds of target genes for each miRNA. Therefore, the number of false-positives is large, making it difficult to identify targets of real interest. More recent expression-based methods incorporate mRNA and miRNA expression data to predict functional miRNA-mRNA interactions. Although useful, most of the algorithms utilize the pairwise negative correlation coefficient to measure the interaction. Focusing exclusively on linear inverse correlations, they may undermine the many to many miRNA-mRNA relationships.

**Figure 1:**
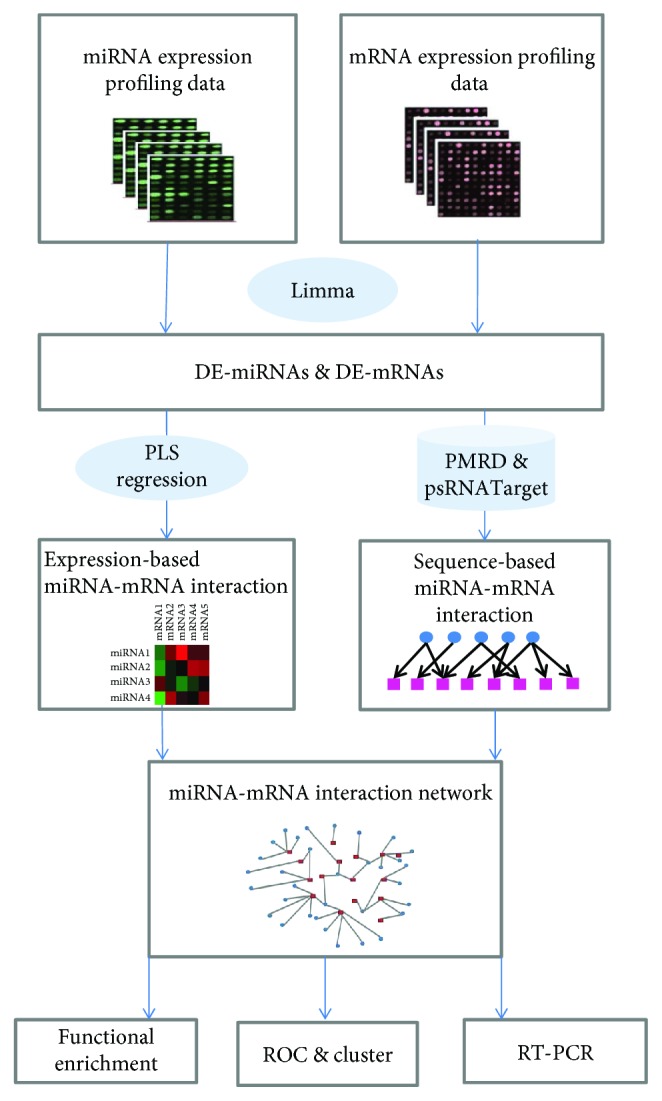
Flow chart of the general procedure of this study.

The aim of the present study was to detect accurate miRNA-mRNA associations in rice under drought stress. We proposed an integrative approach that combines PLS regression and sequence-based target prediction to identify potential miRNA and their targets that are important in drought response. We constructed a miRNA-mRNA regulatory network, and identified 4 miRNA-mRNA regulatory modules in the network that were associated with drought response. Furthermore, we evaluated the expression level and anticorrelation of the putative miRNA and targets. Functional analysis uncovered some drought-responsive canonical pathways that are specifically responsive to drought stress. This report demonstrates the utility of our approach to obtain new insights into posttranscriptional gene regulation at the miRNA level in drought-stressed rice. The schematic diagram of the procedures is given in Figure 1.

## 2. Materials and Methods

### 2.1. Plant Materials and Stress Treatment

Seeds of the rice cultivar Nipponbare were sterilized with 3% (*v*/*v*) NaClO, rinsed with distilled water, and pregerminated in moist filter paper for 48 h at 28°C. Thereafter, the seeds were transferred to sandy loam soil and grown under controlled conditions (16/8 h photoperiod, day/night air temperature of 25°C/20°C, 60% RH, and light intensity of 250 *μ*mol m^−2^ s^−1^). Four-week-old plants were dehydrated by 20% polyethylene glycol 6000 for 48 h to simulate moderate drought stress of −0.5 MPa osmotic potential [27]. The control group was maintained under well irrigation conditions. Fifteen plants were included in each treatment and control group. After the treatment, the leaf disks (6 mm*ϕ*) were excised from the uppermost rice leaf blades from both stress and control plants and stored in liquid nitrogen. Total RNA was isolated from 400 mg leave tissues by using the mirVana™ miRNA Isolation Kit according to the manufacturer's instructions. Three biological replicates were prepared for microarray analysis.

### 2.2. Microarray Analysis

miRNA microarray (LC Sciences, Houston, USA) contains 4 triplicates of 509 unique miRNA probes of 29 species in Sanger miRBase Release 19.0. *Arabidopsis thaliana* 5S rRNA and PUC2-20B were used as positive controls. Approximately 5 *μ*g of the total RNA was size-fractionated, and the isolated small RNAs (~300 nt) were 3′-polyadenylated using polyA polymerase (Takara, Dalian, China) and subsequently ligated to Cy5 for fluorescent staining. Hybridization was performed at 34°C for 20 h. The mRNA expression was analyzed on Agilent-015241 Rice Gene Expression 4x44K Microarray (Feature Number version). The microarray raw data were submitted to the NCBI/GEO database and can be found under the accession number GSE99083 (https://www.ncbi.nlm.nih.gov/geo/query/acc.cgi?acc=GSE99083).

### 2.3. Statistical Analysis

Data was processed in R programming environment v3.3.1. Raw expression files were background subtracted with the Normex method. Normalization was performed using the quantile method. Differentially expressed probe sets were identified using empirical Bayes method using the Limma v3.10. Signals with adjusted *P* value < 0.05 (Benjamini-Hochberg) were listed as significant.

### 2.4. miRNA Target Prediction

PMRD (http://bioinformatics.cau.edu.cn/PMRD/) and psRNATarget (http://plantgrn.noble.org/psRNATarget/) were searched for potential target sites. PMRD was searched with default parameters. The maximum expectation of psRNATarget was set as 2.5 to minimize the number of nonauthentic targets, and other settings remained default. Only the targets predicted by both databases were chosen to increase the prediction accuracy.

### 2.5. miRNA-mRNA Interaction Prediction

PLS regression can be used to measure the relationship between a given gene and other genes using their expression profiles [28, 29]. Briefly, consider a dataset of *p* miRNAs and *m* mRNAs for which *N* measurements are recorded. Let *Y*_*i*_ be the expression profile for the *i*th mRNA, 1 < *i* < *p*. PLS first computes a number *v* < *N* of latent factors *t*_*i*_^(*l*)^, called components for each mRNA, which represents the *l*th latent variable out of all orthogonal latent variables (*l* < <*p*), and then a separate regression model is fitted:

(1) $$\begin{array}{c} Y_{i}=\overset{v}{\underset{l=1}{\sum }}\beta_{il}t_{i}^{\left(l\right)}+e_{i}, \end{array}$$

where *e*_*i*_ is an *n*-dimensional vector of residuals.

Ordinary least squares calculate as follows:

(2) $$\begin{array}{c} {\overset{\wedge }{\beta }}_{il}=\frac{t_{i}^{{\left(l\right)}^{T}}Y_{i}}{t_{i}^{{\left(l\right)}^{T}}t_{i}^{\left(l\right)}},\;1\leq l\leq v. \end{array}$$

The overall association score is then given by

(3) $$\begin{array}{c} {\overset{\wedge }{S}}_{ij}=\frac{\sum_{l=1}^{v}\beta_{il}}{\overset{\wedge }{\sigma }\left(x\right)}\overset{\wedge }{\sigma }\left(y_{i}\right). \end{array}$$

PLS regression was run with 30 deregulated miRNAs and 329 mRNAs to calculate the association scores for each gene pair and recorded in a 329 × 30 matrix.

### 2.6. Significance Evaluation

Repeated bootstrapping (1000 times) was performed to assess the statistical significance of the miRNA-mRNA relationship (*b*_*ij*_). The interactions with *P* value < 0.01 and FDR < 0.05 were regarded as significant.

### 2.7. Network Construction

miRNA-mRNA interaction network was built and visualized with Cytoscape v3.4.0.

### 2.8. Functional Enrichment Analysis

The targets of DE-miRNAs under drought were mapped to GO and KEGG for functional enrichment using the DAVID Bioinformatics tool [30]. Terms with Benjamini-corrected *P* value < 0.05 were listed as significant.

### 2.9. Expression Validation

We employed RT-qPCR, hierarchical clustering, and ROC analysis to validate the expression levels of drought-responsive rice miRNAs.

PrimeScript one-step miRNA cDNA Synthesis Kit (Takara) was used for reverse transcribing mature miRNAs according to the manufacturer's instructions. Universal primers were provided with the kit, and the miRNA-specific primers are given in Supplementary Table S1. SYBRH Premix Ex TaqII (Takara, Japan) was used to quantify mature miRNAs. The PCR amplification parameter was set as 94°C for 2 min, then 40 cycles of 95°C for 10 s, 60°C for 30 s, and 72°C for 1 min. The melting curves were adjusted as 95°C for 1 s and 40°C for 2 min. The 2-DDCT method was used to analyze the relative expression changes. The rice UBC and U6 RNAs were used as internal standards. ef1a was used as an internal control.

Hierarchical clustering was performed with Cluster 3.0. Heat map was visualized with Java TreeView 1.1.6. ROC (receiver operating characteristic) analysis was performed by SPSS 16.0.

### 2.10. RNA Ligase-Mediated 5′ RACE

Total RNA was separately extracted using TRIzol reagent and subjected to RLM-5′ RACE with 5′-Full RACE Kit (Takara, Japan) following the manufacturer's instructions. Briefly, 10 *μ*g of total RNA was ligated to the RNA adapter followed by reverse transcription using the Oligo (dT) primer and superscript reverse transcriptase. PCR was performed with 5′ adaptor primers and 3′ gene-specific primers (Supplementary Table S2). The final PCR product was purified on 2% agarose gel and cloned into a pEASY-T1 Vector, and plasmid DNA was sequenced by Huada Biotech Co.

## 3. Results and Discussion

Dwindling water resources frequently challenge the quality and quantity of rice and cause high economic losses [14]. miRNAs are central to posttranscriptional gene regulation in a wide range of cellular processes including stress responses [6, 31]. Over the past decade, several drought-related miRNA families in rice have been identified by either microarray hybridization or RNA-seq [20, 21, 32]. Stress-responsive miRNAs regulate their target mRNAs by targeting the gene transcripts most of which are stress-associated functional proteins, e.g., transcription factors or hormones involved under drought stress [25]. Therefore, we hoped to uncover significant miRNA-mRNA relationships that are specifically responsive to drought stress [26].

In this study, a multistep approach combining microarray miRNA and mRNA expression profile and bioinformatics analysis was adopted to identify the drought-responsive miRNA-mRNA regulatory network. First, we simultaneously analyzed miRNA and mRNA expression profiles in the same rice leaf sample and identified 76 differentially expressed miRNAs and 2916 mRNAs in drought-stressed samples. Secondly, 13 deregulated miRNAs and their 58 target mRNAs (69 miRNA-mRNA pairs) were identified by a combination of PLS regression approach and sequence-based target prediction. Their differential expression and regulation were further validated experimentally by RT-qPCR and RLM-5′ RACE.

Most of the existing algorithms for miRNA target prediction are based on the sequence complementarity, and the results vary widely. A rule of thumb to address this problem is to intersect the results of multiple prediction algorithms to reduce the false-positive. However, this procedure improves accuracy at the cost of many bona fide targets and therefore leads to poor sensitivity. The research pipeline in this study combines the sequence features and the expression of miRNAs and mRNAs simultaneously. Thus, our method precludes artifacts and improves prediction accuracy, thereby facilitating a more targeted experimental validation.

### 3.1. Identification of Drought-Responsive miRNAs and mRNAs in Rice

Microarray-based miRNA and mRNA expression profiles for *Oryza sativa* under/without drought stress were simultaneously investigated. As a result, 76 miRNAs (FDR < 0.05) were identified to be differentially expressed between drought-stressed and control leaves, corresponding to 38 rice miRNA families. The list of 76 differentially expressed miRNAs is provided in Supplementary Table S3. Of these, 9 were upregulated and 67 were downregulated. Using the same criteria, 2916 genes were identified to be differentially expressed.

### 3.2. PLS Reveals Significant miRNA-mRNA Associations

We first filtered possible miRNA target genes using miRNA target databases PMRD [33] and psRNATarget [34] and obtained 346 miRNA-mRNA pairs among 30 DE-miRNAs and 329 DE-mRNAs.

Next, we used the PLS regression method to remove false-positive discoveries among the 346 miRNA-mRNA pairs in order to find the most likely miRNA-mRNA associations.

The 30 DE-miRNAs were input as independent variables while the 329 DE-mRNAs as dependent variables for PLS regression. The association score *b*_*ij*_ was calculated for each miRNA-mRNA pair, and repeated bootstrapping (1000 times) was subsequently performed to identify significant associations. Repeated bootstrapping (1000 times) was performed to assess the statistical significance of the miRNA-mRNA relationship. The interactions with *b*_*ij*_ > 0.8, *P* value < 0.01, and FDR < 0.05 were regarded as significant.

This filtering step reduced considerably the number of potential candidate interactions, resulting in 69 significant miRNA-mRNA pairs among 13 DE-miRNAs and 58 DE-mRNAs, as listed in Table 1. Among them, 11 miRNAs were significantly downregulated and 2 miRNAs were upregulated. The maximum number of targets was 11 for osa-miR156b-3p.

Among the 13 differentially expressed miRNAs, some are known to respond to drought, including miR156b-3p [21], miR159a.2 [21], miR164c [35], miR169d [32], miR172d-5p [21], miR319a-3p [21], miR395a [21], miR396g [21], miR444b.1 [36], miR528-3p [24], and miR529a [21]. Two novel miRNAs (miR812q and miR1430) are reported for the first time to be involved in drought stress in rice, and their role remains unknown.

The regulatory edges in the miRNA-mRNA network well reflect the existing evidence described in the literature, indicating a high predictive value of our integrative method in identifying genuine miRNA-mRNA interactions.

Prominent drought-responsive miRNA targets were found to encode well-known stress-associated transcription factors or kinases. For example, MADS-box transcription factors targeted by miR444b.1. participate in diverse stress conditions, such as cold stress [37]. SBP-box transcription factor family members targeted by miR156b-3p and miR529a are involved in vegetative phase change induced by various environmental stimuli [38].

ATP-sulfurylase targeted by miR395a is involved in sulfate assimilation and allocation. miR169d targets NFYA regulates the expression of drought-responsive genes, e.g., GT and POD, and is crucial for plant adaptation to environmental stress [39]. miR319a targets two TCP family members (TCP7, 14) that inhibit cell proliferation as well as MYB4 and MYB59 mRNAs. miR164c targets NAC transcription factors, e.g., NAC22 and CUC, which determines the cellular levels of free auxin [40]. miR812q targets CIPK10, which interacts with CBL to trigger the CBL-CIPK-signaling pathway-modulating environmental stress tolerance [41].

### 3.3. Analysis of Drought-Responsive miRNA Coregulatory Network

Using the statistically significant PLS association score between the miRNA and mRNA, we built interaction networks out of the significant DE-miRNAs and their DE-mRNA targets. Edges are formed between miRNA-mRNA pairs with *b*_*ij*_ > 0.8. The resulting miRNA-mRNA regulatory network is essentially a bipartite graph including two disjoint sets of vertices, miRNAs and mRNAs. The network consists of 69 regulations between 13 miRNAs and 58 mRNAs.

**Figure 2:**
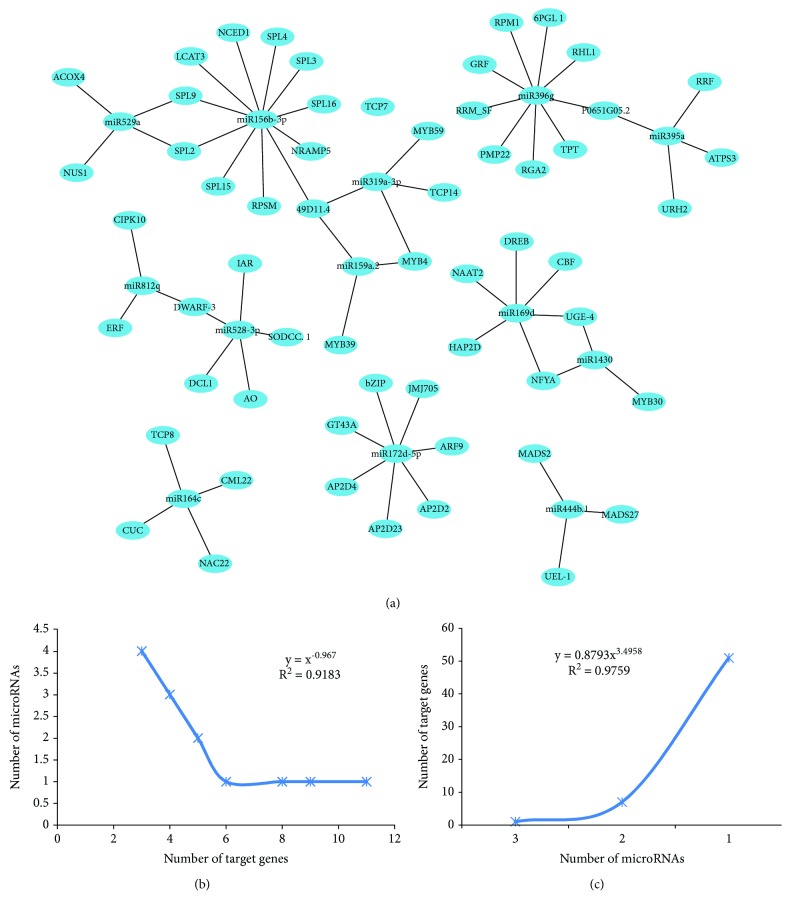
The layout of the miRNA-mRNA network and its structural features. (a) miRNA-mRNA network constructed by PLS. (b) Out-degree distribution of the miRNA-mRNA network. (c) In-degree distribution of the miRNA-mRNA network.

Then we analyzed the out-degree and in-degree distribution of the network (Figures 2(b) and 2(c)). On average, each miRNA has 3–5 outgoing edges and is loosely connected with each other, with a few exceptions such as the highly conserved miR156b-3p with 11 target genes, most of which belong to the SPL family, and the miR172d-5p with 3 target genes belonging to APD family. The out-degree distribution fits the power law with a slope of −0.8399 and *R* ^2^ = 0.9618. The in-degree distribution reveals an exponential distribution with an exponent of −0.967 and *R* ^2^ = 0.9183. It is obvious that as with many biological networks, the miRNA-mRNA network is not random but features a core set of organizing principles in structure.

### 3.4. PLS Reveals miRNA-mRNA Coregulatory Modules

The PLS regression method successfully models the complex interrelationship of multiple miRNAs regulating one mRNA and identified miRNA-mRNA coregulatory modules, i.e., groups of miRNAs that regulate groups of mRNAs in concert. As shown in Figure 2(a), miRNAs regulate at least 3 targets, and 13.8% of mRNAs are coregulated by multiple miRNAs, indicating target multiplicity and miRNA cooperation in the drought response of rice.

One mRNA, 49D11.4 is common target of 3 significantly downregulated miRNAs, miR156b-3p, miR159a.2, and miR319a-3p. The coregulation of miR159 and miR319 was expected, since mature miR159 and miR319 have sequence similarity at 17 out of 21 nucleotides and belong to the same family [42]. The miRNAs within the same family are often derived from recent duplication events and functionally related. Likewise, miR529a showed 14 nucleotide similarities with miR156a-3p and is a shared common target of the SPL family (SPL2, 9) [43]. Moreover, miR1430 is similar to miR169d in sequence and shares the target of NFYA and UGE4 [44].

On the other hand, miRNAs within the same families also have exclusive targets. As shown in Figure 2(a), miR159 and miR319 share MYB targets, but miR159 fails to guide cleavage of TCPs as miR319. This case argues that a single nucleotide difference is sufficient to dictate miRNA target binding.

Interestingly, coregulation occurs also between phylogenetically unrelated miRNAs, i.e., miRNA belonging to different families, e.g., DWARF-3 by miR528-3p and miR8121 and P0651G05.2 by miR396g and miR395a, representing a stress-specific functional redundancy.

Coordinate miRNA regulation has previously been demonstrated in Drosophila and mammals. let-7/lin-4 is the first miRNA pair to be experimentally verified in Drosophila [45]. miR-375/miR-124/let-7b were found to jointly regulate Mtpn in mammals [46]. In this study, we provide evidence for miRNA synergism in drought-stressed rice. It is proposed that synergistic gene regulation is less dependent on miRNA abundance so that limited miRNAs are able to regulate more genes. Moreover, through synergism, miRNAs may exert reinforced repression effects and therefore enhance their regulatory efficacy. The identification of coregulatory miRNA modules would facilitate researchers to make more-informative choices of multiple mutant combinations to circumvent redundancy of miRNA regulation under drought stress.

### 3.5. Hierarchical Clustering and ROC Analysis

**Figure 3:**
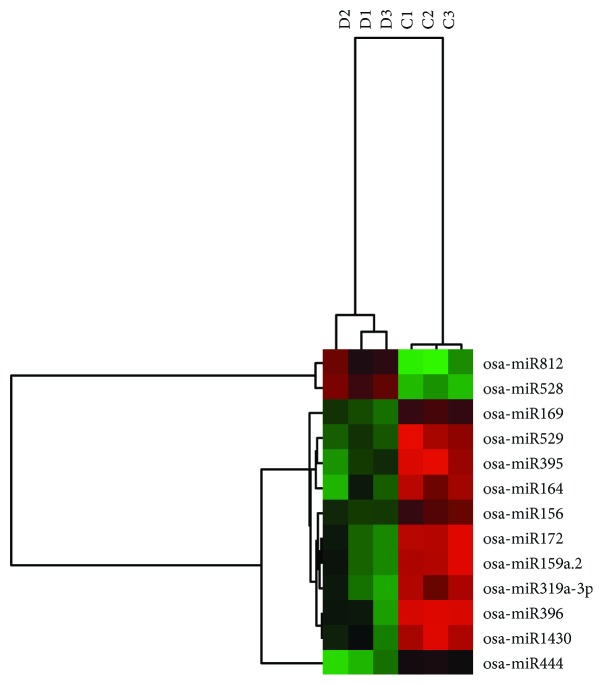
Heat map of the 13 DE-miRNAs in drought-stressed and control rice leaves.

Hierarchical clustering and ROC were performed to validate the differential miRNA expression profiles. A heat map is illustrated in Figure 3.

The AUC (areas under the ROC curve), sensitivity, specificity, and accuracy for the 13 candidate miRNAs are listed in Table 2, and the ROC curves are provided as Supplementary Figure S1. ROC is a way of evaluating the performance of binary classifiers in classifying two classes, e.g., drought vs. control. A ROC curve is a plot of the true positive rates (sensitivity) vs. false-positive rates (1 − specificity). ROC curves can be used to evaluate the quality of classifiers for different discrimination thresholds. The closer the ROC curve to the upper left corner, the greater the AUC and the better the discrimination accuracy. The AUC for the 13 miRNAs are 0.870–0.986, with an average accuracy of 80.76%. The results showed that the candidate DE-miRNAs are able to differentiate drought-stressed samples from the controls. ROC analysis demonstrated that the miRNAs identified in this study are both sensitive and specific in sample discrimination, indicating that they are authentic miRNAs under drought stress.

### 3.6. RT-qPCR Validated the Expression Trends of Drought-Responsive miRNAs and Coregulated Target Genes

We conducted RT-qPCR analysis to validate the expression level of 13 drought-responsive miRNAs and their anticorrelation with coregulated target genes. The results were consistent with the microarray data (Supplementary Figure S2). The relative expression trends of all of the 13 miRNAs were confirmed as differentially expressed under drought stress and were negatively correlated with their target genes.

### 3.7. RLM-5′ RACE Confirmed Direct Targets of Coregulatory miRNAs

To confirm the direct targets of coregulatory miRNAs, we performed RLM-5′ RACE to determine the exact miRNA cleavage sites on target genes. Two mRNAs (DWARF-3 and P0651G05.2) that are coregulated by miRNAs which do not belong to the same family were chosen for verification.

**Figure 4:**
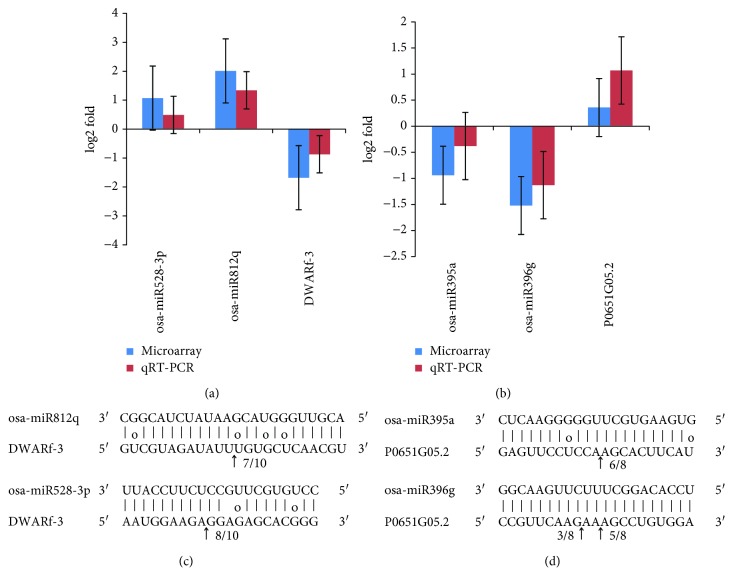
(a) Expression level of coregularory miR528-3p/miR812q and their target DWARF-3 by microarray and RT-qPCR. (b) Expression level of coregularory miR395a/miR396g and their target P0651G05.2 by microarray and RT-qPCR. The expression level is expressed as the mean fold changes of 3 replicates. Error bars depict the standard error (*n* = 3). (c) miR528-3p and miR812q cleavage sites on DWARF-3 identified by RLM-5′ RACE. (d) miR395a and miR396g cleavage sites on P0651G05.2 identified by RLM-5′ RACE. Arrows represent the 5′ termini of mRNA fragments, and the numbers beside them denote the cleavage frequency of cloned PCR products at the exact miRNA cleavage sites. The vertical dashes indicate matched RNA base pairs and circles represent GU mismatch whereas no dashes represent other types of mismatch.

After sequencing the RLM-5′ RACE PCR products, we found that both DWARF-3 and P0651G05.2 had specific cleavage sites in the complementary miRNA sequences (Figures 4(c) and 4(d)). DWARF-3 was cleaved by miR528-3p and miR812q at positions 776 and 1150, respectively. Both cleavage sites are located at the positions 12–13 of miRNAs from the 5′ end. P0651G05.2 can be cleaved at position 486 by miR395a. The cleavage site was mapped to the paired miR395a sequence between the nucleotides 10 and 11. Moreover, P0651G05.2 could be simultaneously cleaved at the two sites by miR396g. The cleavage sites are located between nucleotides 10 and 11 and between 12 and 13, indicating that the cleavage sites of miR396g may be diverse.

The results of RLM-5′ RACE generally confirmed the coregulation of miR528-3p and miR812q on DWARF-3, as well as the coregulation of miR395a and miR396g on P0651G05.2, indicating a high reliability of the PLS analysis.

miR812q and miR528-3p were found to target auxin receptor F-box protein DWARF-3, which is essential for auxin-mediated signaling and plant development [47]. When auxin binds to DWARF-3, it promotes the ubiquitination and degradation of auxin transcriptional repressors, thereby activating auxin-responsive genes [48]. As a coreceptor for phytohormone, e.g., strigolactones and polar auxin, DWARF-3 has a pleiotropic effect on stalk height, leaf angle, and root growth in plants [49, 50]. Under drought stress conditions, upregulated miR812q and miR528-3p would decrease the expression of DWARF-3, and the miRNA-mediated DWARF-3 suppression would lead to attenuation of auxin signaling and inhibition of lateral root growth. We assume that the miR528-3p/miR812q-DWARF-3 coregulatory module might play a central role in development-related gene transcription and plant adaptation to drought stress.

In this study, we only validated two targets, DWARF-3 and P0651G05.2 by RACE experiment, because they are coregulatory hub nodes in the miRNA target network. Degradome sequencing is also a primary and effective tool to prove the miRNA targets. It can directly detect cleaved miRNA targets without predictions or overexpression. Degradome sequencing has been performed in rice by several studies and revealed endogenous small RNA targets under specific tissues or treatments [51, 52]. In the future, a large-scale target validation can be performed based on degradome sequencing data.

### 3.8. Functional Annotation of Drought-Stressed miRNAs

MiRNAs and their predicted target mRNAs were mapped to Gene Ontology and KEGG pathways to explore their biological significance.

**Figure 5:**
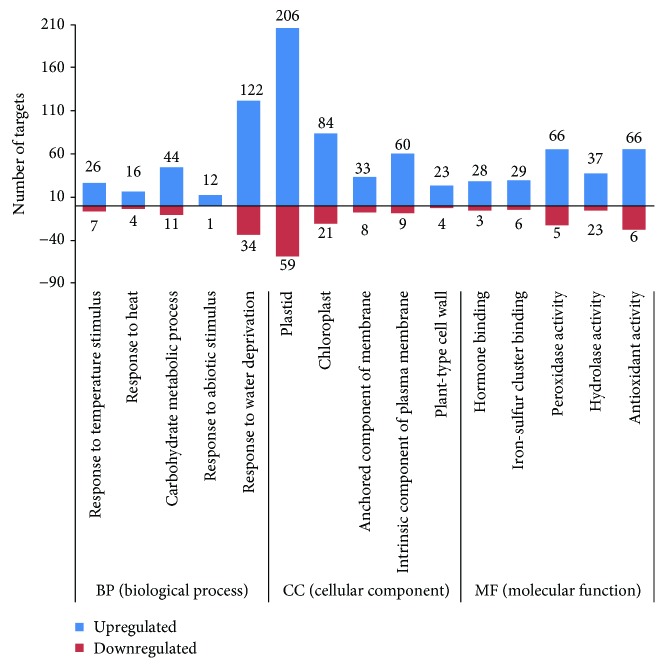
Enriched GO terms of drought-responsive miRNA targets.

According to GO enrichment, the target of drought-responsive miRNAs belonged to five important biological processes (BP): response to temperature stimulus, response to heat, response to abiotic stimulus, carbohydrate metabolic process, and response to water deprivation. Enriched molecular function (MF) terms included hormone binding, iron-sulfur cluster binding, hydrolase activity, peroxidase activity, and antioxidant activity. Plastid, chloroplast, anchored component of membrane, intrinsic component of plasma membrane, and plant-type cell wall were most significant among the cellular components (CC). A detailed list of the enriched GO terms is given in Figure 5.

KEGG pathway analysis unraveled significant activation of hormone signaling, lipid and carbohydrate metabolism, and antioxidant defense under drought. The full list of significant pathways (adjusted *P* value < 0.05) and the number of differentially expressed genes enriched in each pathway are represented in Supplementary Table S4.

#### 3.8.1. miRNA-Regulated Phytohormone Signaling

Plant hormone signal transduction represented the most significantly enriched pathway with 52 genes differentially expressed under drought. A number of miRNA-regulated phytohormones are known to participate in plant responses to abiotic stress, e.g., auxin, salicylic acid, ethylene, GAs, jasmonate acid, and abscisic acid.

Here we found that the expression of key genes in auxin synthesis and the ABA signaling pathway was induced under drought stress. For example, IAR1 transcripts are downregulated by increased miR528 under drought. IAR1 regulates auxin homeostasis by hydrolyzing an inactive form of auxin to release bioactive auxin [53]. The decreased IAR1 expression and hence decreased auxin level leads to the inhibition of adventitious rooting under drought. Several genes in the ABA signaling pathway were also activated under drought. ABA is a plant stress hormone produced under environmental stresses [54]. It is an apocarotenoid derivative produced via oxidative cleavage of carotenoids. Also, we found drought stress increases carotenoid synthesis, which suggests that carotenoid synthesis and the ABA signaling were cooperatively involved in the drought stress response.

#### 3.8.2. Lipid and Carbohydrate Metabolism

Pathway analysis unraveled significant reduction of starch-sucrose metabolism. This includes a variety of sucrose synthases, amylase, and starch branching enzymes. Decreased starch and sucrose concentration is an adaptive feature under drought stress [55], since it allows plants to divert assimilates and energy intended for growth into protective responses for survival [54].

In the context of fatty acid metabolism, *α*-linolenic acid metabolism was significantly induced under drought. *α*-linolenic acid is an unsaturated fatty acid in membrane lipids [56]. It enhances plant tolerance by maintaining membrane fluidity during stress [57].

#### 3.8.3. miRNA-Mediated Antioxidant Defense

The immediate response of plants to stress is accumulation of reactive oxygen species (ROS). To remove the excessive ROS, the cell produces an antioxidative enzyme system, e.g., POD, AO, and SOD that serve as ROS scavengers.

**Figure 6:**
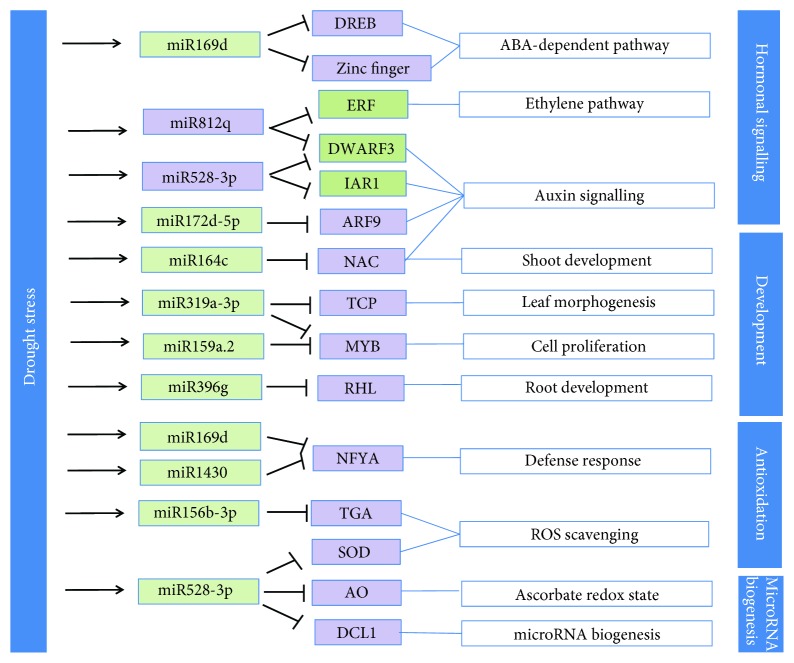
Schematic diagram of miRNA-regulated metabolic processes in rice under drought stress. Under drought stress, rice responds with the following physiological changes: (1) activating hormonal signaling pathways, (2) immobilizing fatty acid and downregulating starch and sucrose metabolism, (3) producing antioxidant compounds for detoxification, and (4) inhibiting miRNA biogenesis.

In this study, we found that the production of ROS scavengers is regulated by miRNAs, as illustrated in Figure 6. For example, miR528 targets genes coding ascorbate oxidase, a central component of the hydrogen peroxide scavenging network. Ascorbate metabolism reduces oxidative damage by maintaining the ascorbate redox state in the form of apoplastic dehydroascorbate [58]. In addition, miR528 targets SOD transcripts, which catalyzes the dismutation of O_2_^−^ and alleviates the damage from ROS. Drought also induces key enzyme genes involved in biosynthesis of several secondary metabolites, such as isoflavonoids and carotenoids which mitigate diverse stress in plants.

A simple model was constructed according to the function of miRNA and target genes under drought stress (Figure 6).

## 4. Conclusion

In conclusion, this study employed a multiple regression model for integrating paired miRNA and mRNA expression profiles. The integrated approach uncovered high-confidence reciprocally expressed miRNA target pairs, supporting the existence of a coregulatory network triggered by drought stress. Further validation of these hub miRNAs as coregulatory nodes may facilitate the development of novel molecular markers for breeding of rice cultivars with enhanced drought tolerance. In-depth analysis of coregulatory networks will reveal the regulatory role of miRNAs in a broader range of a posttranscriptional network under drought stress.

## Figures and Tables

**Table 1:** Drought-responsive miRNAs and their target genes predicted by PLS.

miRNA	Family	Response	Target no.	Targets predicted by PLS	Literature
osa-miR1430	MIR169	Down	3	MYB30, NFYA, UGE-4	
osa-miR156b-3p	MIR156	Down	11	49D11.4, LCAT3, NRAMP5, RPSM, SPL15, SPL16, SPL2, SPL3, SPL4, SPL9, NCED1	[21, 24]
osa-miR159a.2	MIR159	Down	3	49D11.4, MYB4, MYB39	[21, 24]
osa-miR164c	MIR164	Down	4	CML22, CUC, NAC22, TCP8	[35, 36]
osa-miR169d	MIR169	Down	6	CBF, DREB, HAP2D, NAAT2, NFYA, UGE-4	[32]
osa-miR172d-5p	MIR172	Down	7	AP2D2, AP2D23, AP2D4, ARF9, bZIP, GT43A, JMJ705	[21]
osa-miR319a-3p	MIR159	Down	5	49D11.4, MYB4, MYB59, TCP14, TCP7	[21]
osa-miR395a	MIR395	Down	4	ATPS3, P0651G05.2, RRF, URH2	[21]
osa-miR396g	MIR396	Down	9	6PGL 1, P0651G05.2, PMP22, RGA2, RHL1, RPM1, RRM_SF, TPT	[21]
osa-miR444b.1	MIR444	Down	3	MADS2, MADS27, UEL-1	[36]
osa-miR528-3p	MIR528	Up	5	AO, DCL1, DWARF-3, IAR, SODCC.1	[24]
osa-miR529a	MIR529	Down	4	ACOX4, NUS1, SPL2, SPL9	[21]
osa-miR812q	MIR812	Up	3	CIPK10, DWARF-3, ERF	

**Table 2:** ROC analysis of drought-responsive miRNAs.

miRNA	AUC	Sensitivity	Specificity	Accuracy
osa-miR1430	0.930	0.900	0.900	0.800
osa-miR156b-3p	0.978	0.900	1.000	0.900
osa-miR159a.2	0.908	0.800	1.000	0.800
osa-miR164c	0.902	0.900	0.800	0.700
osa-miR169d	0.945	0.850	0.950	0.800
osa-miR172d-5p	0.930	0.950	0.900	0.850
osa-miR319a-3p	0.950	0.950	0.950	0.900
osa-miR395a	0.957	0.850	1.000	0.850
osa-miR396g	0.913	0.800	0.950	0.700
osa-miR444b.1	0.870	0.900	0.850	0.750
osa-miR528-3p	0.890	0.800	0.900	0.700
osa-miR529a	0.931	0.850	0.950	0.800
osa-miR812q	0.986	0.900	0.950	0.850

## Data Availability

The microarray data used to support the findings of this study have been deposited in the GEO repository (GSE99083).

## Abbreviations

ABA: Abscisic acid
AUC: Areas under the ROC curve
CIPK: Calcineurin B-like protein interacting protein kinase
ERF: Ethylene-responsive element-binding factor
GO: Gene Ontology
GT: Glutathione transferase
KEGG: Kyoto Encyclopedia of Genes and Genomes
NFYA: Alpha subunit of CCAAT binding NFY transcription factors
POD: Peroxidase
RH: Relative humidity
RLM-5′ RACE: RNA ligase-mediated 5′ rapid amplification of cDNA ends
ROC: Receiver operating characteristic
SOD: Superoxide dismutase
SPL: Squamosa promoter binding protein-like.

## Supplementary Materials

Supplementary 1 Table S1: oligonucleotide primers used in RT-qPCR.

Supplementary 2 Table S2: the specific primer sequences for RLM-5′ RACE of target genes.

Supplementary 3 Table S3: differentially expressed miRNAs under drought stress.

Supplementary 4 Table S4: enriched KEGG pathways of drought-responsive miRNA targets.

Supplementary 5 Figure S1: ROC curves of 13 drought-responsive miRNAs.

Supplementary 6 Figure S2: validation of drought-responsive miRNAs and coregulated target genes by RT-qPCR. The expression level is expressed as the mean fold changes of 3 replicates. Error bars depict the standard error (*n* = 3).
