# Supplementary material for: Multiple Regression Analysis Reveals MicroRNA Regulatory Networks in Oryza sativa under Drought Stress
Source: Int J Genomics. 2018 Oct 4;2018:9395261. doi: 10.1155/2018/9395261 (PMC6196795; doi:10.1155/2018/9395261)
Supplement: Supplementary 5 — Figure S1: ROC curves of 13 drought-responsive miRNAs. [file 9395261.f5.pptx]

## Slide 1
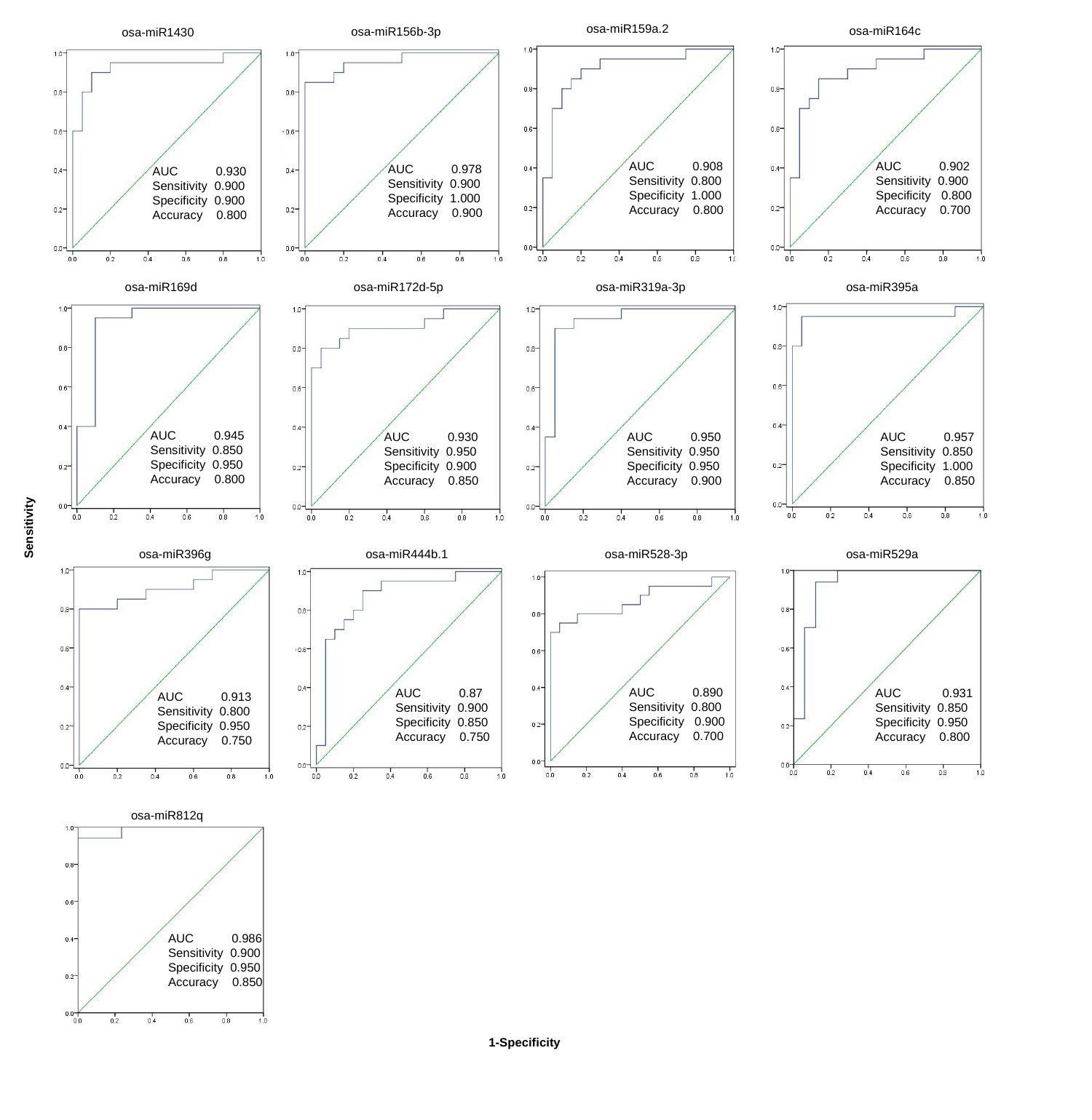

osa-miR159a.2
osa-miR164c
osa-miR156b-3p
osa-miR1430
AUC 0.908
Sensitivity 0.800
Specificity 1.000
Accuracy 0.800
AUC 0.902
Sensitivity 0.900
Specificity 0.800
Accuracy 0.700
AUC 0.978
Sensitivity 0.900
Specificity 1.000
Accuracy 0.900
AUC 0.930
Sensitivity 0.900
Specificity 0.900
Accuracy 0.800
osa-miR169d
osa-miR172d-5p
osa-miR395a
osa-miR319a-3p
AUC 0.945
Sensitivity 0.850
Specificity 0.950
Accuracy 0.800
AUC 0.957
Sensitivity 0.850
Specificity 1.000
Accuracy 0.850
AUC 0.930
Sensitivity 0.950
Specificity 0.900
Accuracy 0.850
AUC 0.950
Sensitivity 0.950
Specificity 0.950
Accuracy 0.900
Sensitivity
osa-miR396g
osa-miR444b.1
osa-miR528-3p
osa-miR529a
AUC 0.890
Sensitivity 0.800
Specificity 0.900
Accuracy 0.700
AUC 0.87
Sensitivity 0.900
Specificity 0.850
Accuracy 0.750
AUC 0.931
Sensitivity 0.850
Specificity 0.950
Accuracy 0.800
AUC 0.913
Sensitivity 0.800
Specificity 0.950
Accuracy 0.750
osa-miR812q
AUC 0.986
Sensitivity 0.900
Specificity 0.950
Accuracy 0.850
1-Specificity
